# Post-Meiotic Intra-Testicular Sperm Senescence in a Wild Vertebrate

**DOI:** 10.1371/journal.pone.0050820

**Published:** 2012-12-03

**Authors:** Attila Hettyey, Balázs Vági, Dustin J. Penn, Herbert Hoi, Richard H. Wagner

**Affiliations:** 1 Department of Integrative Biology and Evolution, Konrad Lorenz Institute of Ethology, University of Veterinary Medicine, Vienna, Austria; 2 Lendület Evolutionary Ecology Group, Plant Protection Institute, Centre for Agricultural Research, Hungarian Academy of Sciences, Budapest, Hungary; 3 Behavioural Ecology Group, Department of Systematic Zoology and Ecology, Eötvös Loránd University, Budapest, Hungary; University of Melbourne, Australia

## Abstract

There is growing interest in sperm senescence, both in its underlying mechanisms and evolutionary consequences, because it can impact the evolution of numerous life history traits. Previous studies have documented various types of sperm senescence, but evidence of post-meiotic intra-testicular sperm senescence in wild animals is lacking. To assess such senescence, we studied within-season changes in sperm motility in the common toad (*Bufo bufo*), where males produce all sperm prior to the breeding season. We found that males exposed to experimentally induced re-hibernation at the start of the breeding season, that is to experimentally lowered metabolic rates, stored sperm of significantly higher motility than males that were kept under seminatural conditions without females throughout the breeding season. This finding indicates that re-hibernation slows normal rates of sperm ageing and constitutes the first evidence to our knowledge of post-meiotic intra-testicular sperm senescence in a wild vertebrate. We also found that in males kept in seminatural conditions, sperm motility was positively related to the number of matings a male achieved. Thus, our results suggest that post-meiotic intra-testicular sperm senescence does not have a genetically fixed rate and may be modulated by temperature and possibly by mating opportunities.

## Introduction

Senescence, which is reduced survival or fertility with increasing age, has been a fundamental problem in evolutionary biology for decades [1]–[4] and there is growing interest in sperm senescence [5]–[6]. Sperm senescence can reduce sperm quality, which in turn can lower fertilization success and offspring viability, resulting in important consequences for individual fitness [6], [7]. Senesced sperm can be the result of two contrasting processes: senescence before and after meiosis. Pre-meiotic sperm senescence is the result of damage accumulated in diploid somatic and germ-line cells with advancing male age. The proximate causes include the accumulation of deleterious mutations and a shortening of telomeres in male germ-line cells due to frequent mitosis, the degeneration of nurse cells and other accessory tissue, and a decline in androgen levels or receptor activity [8], [9]. Pre-meiotic sperm senescence results in older males having lower quality sperm. Post-meiotic sperm senescence progresses during the life of the individual haploid gamete and can occur both during sperm storage within the male and, after sperm transfer, outside of the male in the fertilization environment or in the sperm storage organ of the female. Post-meiotic sperm senescence is caused by various factors, including toxic residues, free radicals and osmotic and thermodynamic stress [10]–[13]. Damage is done to chromatin, mitochondria or membranes, and accumulates quickly due to high metabolic activity, while repair is limited due to relatively little cytoplasmic content of the sperm cell [14], [15]. Post-meiotic sperm senescence causes individual sperm that had undergone meiosis earlier to be of lower quality.

There is substantial evidence of various kinds of sperm senescence occurring in insects, some domestic animals (birds and mammals) and humans, but studies of these model organisms may not be appropriate for understanding sperm ageing in wild vertebrates due to large differences in life histories, human-induced artificial selection for early maturation and high reproductive output, and an artificial environment containing (hormonal) pollutants [16]. We know of no previous reports on post-meiotic intra-testicular sperm senescence in wild vertebrates [6], [17], even though this form of sperm senescence may have several significant evolutionary consequences, such as affecting mating preferences of females [14], [18]–[21], habitat choice and reproductive behavior of males [22]–[24] and timing of reproductive activities in both sexes [25]. Species in which the spermatogenic cycle is discontinuous and sperm production ceases before the onset of the reproductive season provide excellent model systems for examining post-meiotic intra-testicular sperm senescence. In such species ageing sperm do not become diluted by freshly produced sperm in the testes, which maximizes the detectability of potential effects of post-meiotic sperm senescence on sperm quality, without the need of tracking individual spermatozoa. In males of some anuran amphibians, and of common toads (*Bufo bufo*) in particular, there is no sperm replenishment during the reproductive season and males can only use the sperm they accumulated before the onset of the reproductive period [26]–[28].

We investigated within-seasonal changes in sperm quality of male *B. bufo* and expected to find evidence of post-meiotic intra-testicular sperm senescence. We studied sperm quality in isolation from ova because patterns in fertilization success or offspring viability can be confounded by variation in female gamete quality, maternal environmental effects, maternal genetic effects and, most importantly, by variation in the compatibility between male and female gametes [29]–[31]. We tested for signs of pre-meiotic sperm senescence and of a rarely investigated interaction between pre-meiotic and post-meiotic sperm senescence [32], [33]. We also studied potential effects of the presence of females on the rate of post-meiotic sperm senescence. Previous studies were concerned with species exhibiting continuous sperm production, where an increase in motility of stored sperm after ejaculation could be explained by dilution (in the case of sperm mixing) or removal (in the case of sperm stratification) of senesced sperm (for references see [6]), whereas in our model species, where sperm production ceases long before the onset of reproductive activities [26]–[28], any effect of female presence or of the act of repeated matings on sperm motility would clearly suggest the presence of physiological effects on the rate of post-meiotic intra-testicular sperm senescence.

## Materials and Methods

We collected *B. bufo* males from a population in the Pilis Mountains, Hungary (47°42′N, 19°02′E) at the start of the breeding season in 2008. We measured snout to vent length (SVL) with a plastic ruler (to the nearest 1 mm) and used it as a measure of body size. We also weighed animals with a digital scale (to the nearest 0.1 g). Over the course of one night, we collected 100 males and released the three largest, the three smallest and 46 medium-sized males to obtain a sample of 48 males containing males of varying sizes while excluding extreme-sized individuals.

We randomly assigned males to three treatments, each comprising 16 individuals. Males assigned to treatment 1, from here on referred to as ‘re-hibernated males’, were immediately transported to the laboratory where they were stored individually in opaque plastic boxes at 5°C. Anurans are inactive and in a hibernation-like state when kept at 5°C in the dark. Hibernation has been shown to slow ageing-related processes in vertebrates in general [34], [35] and we found that this procedure slowed post-meiotic sperm senescence in our study species (see Results), presumably due to a close relationship between environmental temperature and metabolic rate in the poikilothermic amphibians [36], and a severe decrease of physiological activities during hibernation [37]. A comparison between this treatment, where the rate of senescence was lowered, and the other two treatments, where senescence could progress normally, thus, allowed us to look for signs of post-meiotic sperm senescence. Males assigned to treatment 2 and 3 were kept individually under semi-natural conditions in large plastic containers (90 cm in diameter, 80 cm deep) placed outdoors and holding 15 cm (100 litres) of pond water. Males in treatment 2 were deprived of females to allow us to study potential changes in sperm quality due to post-meiotic intra-testicular sperm senescence over the course of the reproductive season. Males in treatment 3 were repeatedly offered gravid females for mating, to assess potential changes in sperm quality due to repeated matings. From here on, we refer to males in treatment 2 as “unmated males” and males in treatment 3 as “mated males”.

We collected females from the same pond as males, and from two nearby ponds. After capture, we housed females in large plastic boxes (1 m×1 m, 40 cm high) filled with moistened leaves until we used them in the experiment. Once we placed females into experimental containers holding a male, we monitored trials every 2 hours. When a pair completed egg-laying, we removed the egg-string and the spent female and, one day later, placed a new, gravid female into the experimental container. There was wide among-pair variation in the interval between the formation of amplexus and the end of egg-deposition (range: 8–168 hours), allowing males to mate for a varying number of times (one male mated once, five males mated twice, seven males mated three times and three males mated four times). We terminated the experiment 300 hours after commencement, approximately 130 hours after introducing the last available gravid female into a trial. One pair was still in amplexus (for 150 hours) at termination. The time span of our experiment was comparable to the length of the breeding season in nature, where peak reproductive activity spans about one week, with a few more matings occurring some days before and after that period [27], [38]–[40]. After termination, we released all females and embryos and transported males to the laboratory, where we stored them the same way as re-hibernated males, i.e. individually in opaque boxes at 5°C.

Three, four, five and six days after termination of the first part of the study, we over-anaesthetized three re-hibernated males, four unmated males and four mated males with tricaine (MS-222), removed their testicles and macerated them in 10 ml of reconstituted soft water (RSW, [41]). The release of anuran sperm into water, which is hypoosmotic compared to the intracellular environment, leads to sperm activation [42], [43]. One, 10, 30, 60 and 120 minutes after sperm activation, we took 10 µl samples from the sperm suspensions, further diluted them by adding 40 µl RSW (to enhance consequent readability) and pipetted the resulting 50 µl onto specially prepared microscope slides (for details see [44]). We recorded sperm movements with a microscope-attached video camera at x200 magnification in four different areas of the slides for 30 s each. Subsequently, to obtain estimates of sperm motility, we counted the active and total number of sperm visible on each screen and calculated mean values per sperm suspension per time point. Sperm with undulating tail membranes were considered motile, while those with motionless membranes were considered inactive [45]. Although in amphibians sperm may be re-activated to some degree by molecules diffusing from the egg-jelly [46], [47], we assumed that the decreases in sperm motility we estimated with the above method correlated with permanent reductions in sperm quality. Sperm motility has indeed been shown to affect fertilization success in another externally fertilizing frog species [48] and in other taxa as well [49], [50]. Counts were done blind, without knowing to which treatment the observed male had been assigned. Measurements were taken in the laboratory at a constant temperature of 20°C. While 20°C may be higher than average temperatures experienced in the field, all sperm may be similarly affected and matings have been observed in the field at this water temperature (A. Hettyey, unpublished data).

In a related study [28], we also measured testes mass and counted the number of sperm stored in the testes in the same individuals. There we found that the number of sperm stored in the testes was highest in re-hibernated males (mean±SE; 1.36×10^8^±1.13×10^7^), somewhat lower in unmated males (1.23×10^8^±8.74×10^6^) and lowest in mated males (4.94×10^7^±6.83×10^6^) [28]. Sperm store size did not differ between re-hibernated and unmated males, but mated males had significantly smaller sperm stores than males in the other two treatments (Bonferroni-corrected pairwise comparisons of estimated marginal means; re-hibernated vs. unmated males, *P* = 1; re-hibernated vs. mated males, *P*<0.001; unmated vs. mated males, *P*<0.001) [28]. If males of *B. bufo* produced sperm during the breeding season, we should have observed higher numbers in males kept at ambient temperatures and deprived of females than in re-hibernated males. These results decisively supported the conclusion of a previous study [27] on a lack of sperm production during the reproductive season in this species.

Two re-hibernated males died before dissection, probably due to overcooling, and we could not estimate sperm motility for another two due to logistical difficulties, resulting in a sample size of 44 males available for analysis: 12 re-hibernated males, 16 unmated and 16 mated males. Males did not differ in body size or in body condition (residuals from a regression of body mass on SVL) among the three treatments (one-way ANOVA; body size: *F*_2,41_ = 0.13, *P* = 0.88; body condition: *F*_2,41_ = 0.63, *P* = 0.54) and the distribution of body size was continuous and did not deviate from normality (Kolmogorov-Smirnov test; all males: *Z* = 0.11, *df* = 44, *P*>0.20; re-hibernated males: *Z* = 0.17, *df* = 12, *P*>0.20; unmated males : *Z* = 0.20, *df* = 16, *P* = 0.091; mated males: *Z* = 0.15, *df* = 16, *P*>0.20).

The Közép-Duna-Völgyi KTVF issued the permission to collect samples and conduct the experiment (No. 13369-2/2008). This experiment belongs to a wider study of common toads for which we obtained a general permit from the ‘Munkahelyi Állatvédelmi Bizottság’ (Animal Welfare Committee) of the Eötvös Loránd University, Budapest. The toads were housed in the Ecology laboratory of the Department of Systematic Zoology and Ecology of the Eötvös Loránd University, Budapest, and the animals were treated in accordance with the Hungarian Act of Animal Care and Experimentation (1998. XXVIII. Section 243/1998).

### Statistical Analyses

We conducted four analyses. First, to determine whether post-meiotic intra-testicular sperm senescence occurs and causes reduced sperm quality, we compared sperm motility in re-hibernated males with that of unmated males. We used a linear mixed effects model (LMM), entering individual as a random factor, treatment as a fixed factor and time since sperm activation, body size and body condition as covariates. To avoid potential problems arising when ratios or residuals are used [51], we entered the number of motile sperm as the dependent variable into the model, together with total sperm number as a covariate to control for variation in sperm density. Second, we tested whether the presence of females affects sperm quality by comparing sperm motility in unmated versus mated males using another LMM with the number of motile sperm as the dependent variable, individual as a random factor, treatment as a fixed factor, and time since sperm activation, body size, body condition and total sperm number as covariates. Third, to investigate potential changes in sperm motility over the course of repeated matings, we used another LMM, with the number of motile sperm obtained from mated males entered as the dependent variable, individual as a random factor, time since sperm activation, body size, body condition, the number of matings a given male engaged in and total sperm number as covariates. Fourth, to assess whether varying numbers of matings affected sperm quality or, alternatively, whether males that engaged in repeated matings may simply also have had higher sperm motility, we compared variation in sperm motility between unmated and mated males immediately after sperm activation using Levene’s test of equality of error variances. Similar variation would suggest that motility was not affected by the number of matings, whereas larger variation in mated males would suggest that the varying numbers of matings had an effect on sperm motility and resulted in increased variation among males. Whenever applicable, we entered all two-way interactions into initial models (except interactions with total sperm number, because this variable was only entered to statistically control for variation in sperm density). We applied a backward removal of terms with *P*>0.05 to avoid problems potentially arising from the inclusion of non-significant effects. We obtained statistics for removed variables by re-entering them one by one to the final model [52]. Model residuals were normally distributed in all tests. Statistics were calculated using PASW Statistics 18.

## Results

### Presence/absence of Post-meiotic Sperm Senescence

**Figure 1:**
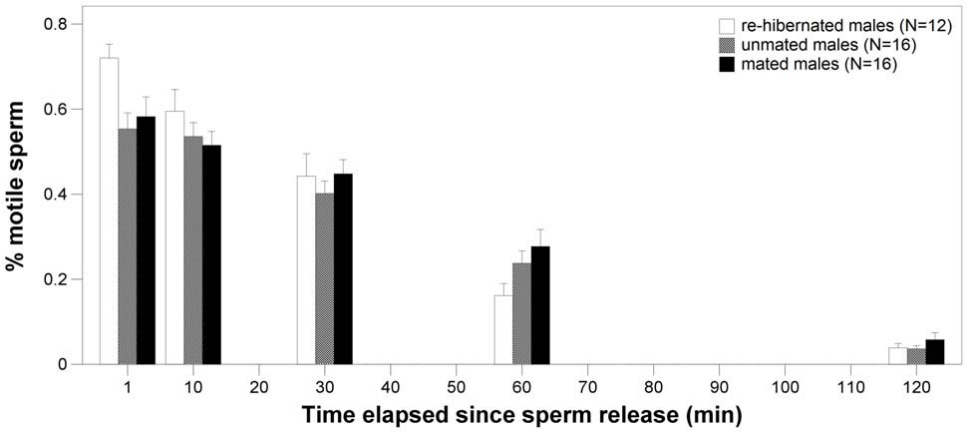
Sperm motility sampled 1, 10, 30, 60 and 120 min after activation in experimentally re-hibernated males (treatment 1), unmated males (treatment 2) and mated males (treatment 3). We show all three treatments to allow direct comparison. Means±SE of untransformed percentage data are shown, sample sizes are indicated.

In the analysis testing for post-meiotic sperm senescence, comparing sperm motility in re-hibernated males with that of unmated males, three variables: treatment, time since sperm activation and total sperm number, had significant effects on the number of motile sperm (LMM; treatment: *F*_1,69.3_ = 15.48, *P*<0.001; time since sperm activation: *F*_1,111.2_ = 277.42, *B* = −0.082, *SE* = 0.008, *P*<0.001; total sperm number: *F*_1,50.5_ = 144.69, *B* = 0.508, *SE* = 0.042, *P*<0.001). Sperm motility decreased over time from 62.51±2.97% (mean±SE) immediately after sperm activation to 3.74±0.6% two hours later (Fig. 1). The main effects of body size and body condition were non-significant (body size: *F*_1,26.6_ = 0.24, *P* = 0.63; body condition: *F*_1,23.5_ = 1.06, *P* = 0.31). The interaction between treatment and body size was marginally non-significant (*F*_1,23.8_ = 4.02, *P* = 0.057; re-hibernated males: *B* = 0.1; *SE* = 0,195; unmated males: *B* = −0.069; *SE* = 0,126), whereas all other interactions involving body condition or body size remained non-significant (all *P*>0.11). The interaction between treatment and time since sperm activation was significant (*F*_1,112.2_ = 14.87, *P*<0.001; Fig. 1), we therefore further analyzed the effects of treatment on the five sampling events separately using General Linear Models [52]. We applied Bonferroni-correction to control for the increased likelihood of reporting false positives due to multiple testing.

Most importantly, we found a significant effect of male treatment on sperm motility immediately following sperm activation (GLM on data from 1 min sampling period; *F*_1,25_ = 16.53, *P* = 0.002): sperm motility was higher by 30% in re-hibernated males (mean % motile sperm±SE: 72.04±3.23%) compared with unmated males (55.36±3.75%). In contrast, sperm motility was comparable between the two treatments at all other sampling intervals (all *P*>0.13). We also found a significant positive relationship between total sperm number and the number of motile sperm 1, 10, 30, and 60 minutes after sperm activation (all *P*<0.001). This relationship was not observed 120 minutes after sperm activation (*F*_1,26_ = 3.83, *P* = 0.31).

### The Effect of Female Presence on Sperm Motility

When testing for an effect of female presence by comparing sperm motility in unmated vs. mated males, we again observed a significant decrease in the percentage of motile sperm over time (LMM; *F*_1,136.3_ = 356.58, *B* = −0.042, *SE* = 0.005, *P*<0.001; Fig. 1), and an effect of treatment (*F*_1,63.7_ = 4.7, *P* = 0.034), where the percentage of motile sperm was on average somewhat lower in unmated males than in mated males (Fig. 1). However, the interaction between time since sperm release and treatment was also significant (*F*_1,127.2_ = 45.68, *P*<0.001; Fig. 1). The effect of total sperm number was again significant (*F*_1,81.8_ = 90.48, *B* = 0.368, *SE* = 0.039, *P*<0.001), whereas main effects of body size and body condition (body size: *F*_1,31.1_ = 1.81, *P* = 0.19; body condition: *F*_1,29.4_ = 0.26, *P* = 0.61), or any interaction terms involving these two variables remained non-significant (all *P*>0.21). Separate analyses conducted on the five sampling events using General Linear Models and subsequent Bonferroni-correction revealed no significant effects of treatment at any single sampling event (all *P*>0.3) and a diminishing effect of total sperm number (immediately at sperm activation and 10, 30 and 60 min: *P*<0.001; at 120 min: *P* = 0.46).

### The Effect of the Number of Matings on Sperm Motility

**Figure 2:**
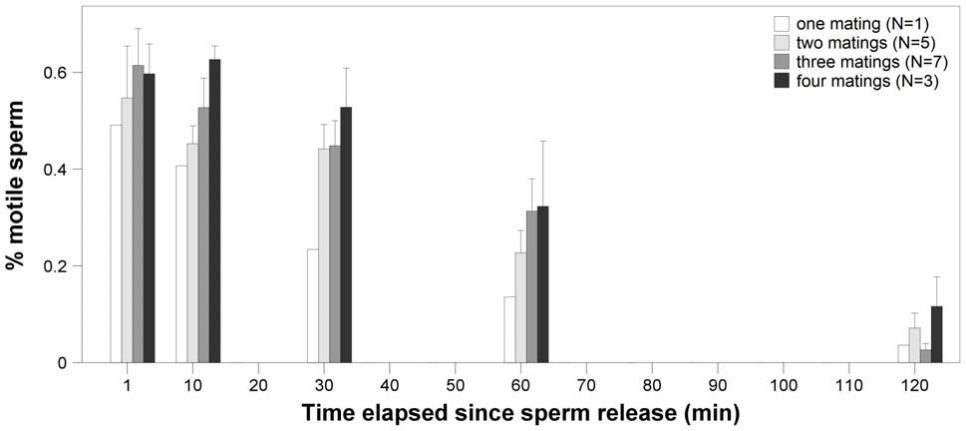
Sperm motility sampled 1, 10, 30, 60 and 120 min after activation in mated males (treatment 3) in relation to the number of times they mated. Means±SE of untransformed data are shown, sample sizes are indicated.

When analyzing potential effects of repeated mating on sperm motility in mated males, sperm motility again decreased with time since sperm activation (LMM; *F*_1,64.9_ = 111.32, *B* = −0.039, *SE* = 0.004, *P*<0.001; Fig. 2). More importantly however, we found a significant positive correlation between sperm motility and the number of matings a given male had performed (*F*_1,16.3_ = 7.92, *B* = 0.93, *SE* = 0.331, *P* = 0.012; Fig. 2). Total sperm number also had a significant positive effect (*F*_1,38.4_ = 101.06, *B* = 0.555, *SE* = 0.055, *P*<0.001), whereas the main effects of body size and body condition were non-significant (body size: *F*_1,11.9_ = 2.14, *P* = 0.17; body condition: *F*_1,11.3_ = 0.68, *P* = 0.43). All two-way interactions were non-significant (all *P*>0.2).

### The Effect of Matings on Among-male Variation in Sperm Motility

When comparing variation among unmated vs. mated males to assess whether it was the presence of females that resulted in the previously described correlation between the number of matings and sperm motility, we obtained significantly lower variation in sperm motility immediately after sperm activation among the 16 unmated males than among the 16 males that had mated a varying number of times (Levene’s test of equality of error variances; *F*_1,30_ = 4.88, *P* = 0.035).

## Discussion

Our study provides the first evidence to our knowledge of post-meiotic intra-testicular sperm senescence in a wild vertebrate. Male toads held under semi-natural conditions over the course of the breeding season (presumably suffering normal rates of sperm senescence) stored sperm with lower motility than males that were re-hibernated at the onset of the season (exhibiting experimentally lowered rates of sperm senescence). Strikingly, this difference was detectable immediately after sperm activation, which is within the time-frame when most fertilizations occur in externally fertilizing animals with simultaneous and syntopic gamete release [53]–[55].

Sperm senescence can have important evolutionary consequences by reducing fertilization success and embryo viability [7]. Females may be selected to avoid males with senesced sperm or to select sperm for fertilization that are still of high quality [14], [18]–[21]. Males on the other hand may be selected to choose certain (i.e., thermal) environments that slow sperm senescence, and to shorten periods of sexual rest by also accepting matings with low-quality females or by discharging aged sperm [22]–[24]. Sperm senescence can also lead to sexual conflict if females avoid fertilizations by senesced sperm by inducing polyandry or via cryptic female choice [56] or if males exhibiting senesced sperm can enforce matings [57]. In species where sperm production ceases before the onset of the reproductive season (e.g., insects (*Plodia interpunctella*): [58]; fishes (*Gasterosteus aculeatus*): [59]; anuran amphibians (*B. bufo*): [27], [28]), females can only avoid fertilizations by senesced sperm by mating early in the season. Thus, as in marine free-spawners [25] post-meiotic gamete senescence may constitute one reason for the evolution of synchronized arrival of males and females at breeding sites and of a short and intensive breeding season, termed ‘explosive breeding’ in anurans [38]. Nonetheless, the opposite evolutionary pathway is equally likely: if there are few opportunities for mating after peak spawning, there is little value in investing into sperm maintenance and repair mechanisms, so that an explosive breeding pattern may result in fast post-meiotic gamete senescence. However, it is important to note, that changes in sperm quality are only expected to have evolutionary consequences if they affect fertilization success or offspring fitness. Further studies are needed to test whether post-meiotic intra-testicular sperm senescence has the potential to affect these parameters.

Given that body size is related to age in animals with indeterminate growth, such as *B. bufo* [60], the observation that sperm motility increased with body size in re-hibernated males, whereas it decreased in unmated males suggests a possible interaction between pre- and post-meiotic sperm senescence. The positive relationship between body size and sperm motility in re-hibernated males may have indicated that older males have higher quality sperm at the onset of the reproductive season than younger males. However, this relationship turned negative by the end of the breeding season, suggesting that sperm of older males senesce quicker than sperm of younger males. A potential explanation is that pre-meiotically senesced sperm may carry more mutations that make them vulnerable to post-meiotic damage (*sensu* [61]). Also, older males may invest more resources into bodily maintenance, which has negative tradeoffs with the production of sperm withstanding post-meiotic stress (*sensu* [62]). However, since the relationship between body size and age is not strong in *B. bufo* and the tendency we observed relied on one data point, this result remains inconclusive. Observations on similar interactions between pre-and post-meiotic sperm senescence are scarce and deserve more attention [6].

We also observed a positive relationship between sperm motility and the number of matings. It is unlikely that this relationship resulted from higher quality males producing higher quality sperm *and* also achieving elevated mating success, because while this explanation would assume no difference in variation between unmated and mated males, variation in sperm motility was significantly higher in mated males. Instead, this result suggests that the presence of several females, or the act of repeated mating, may somehow lead to retarded senescence of sperm. Previous studies have shown that male vertebrates can adjust sperm quality to their social status [63]–[65], female quality [66] and mate availability [67]. It is possible that the presence of potential mating partners or actual matings activated hormonal signals that lowered rates of sperm senescence (e.g., by enhancing local antioxidative enzyme expression [68]). Alternatively, limited resources (energy, extracellular repair mechanisms, anti-oxidants, etc.) may allow better maintenance of fewer sperm. Hypothetically, post-hibernation spermatogenesis may have also resulted in the observed relationship between the number of matings and sperm motility: Males that mated more times may have had a somewhat lower proportion of old sperm, resulting in increased overall motility. However, previous studies showed that in *B. bufo* sperm production ceases before the onset of the breeding season [27], [28], which refutes this hypothesis.

The observed among-treatment differences in sperm motility (see Fig. 1) may partly be explained by a decline in sex-hormone levels over the mating period [69], [70]. However, this interpretation cannot fully explain the observed patterns in sperm motility; males that had mated once or twice showed lower sperm motility than unmated males (males kept without females). Consequently, as in mated males with females present, hormone levels should have been maintained at a higher level, which in turn should have resulted in higher sperm motility relative to unmated males, where females were not present. Also, even though males that mated 3–4 times should have had higher sex-hormone concentrations, their sperm had lower motility than those of males that were re-hibernated. Consequently, changes in sperm motility do not seem to be reversible all together by mating. Thus, the most likely explanation is that sperm senesced over the reproductive season and this process was influenced by the social environment. It remains to be explored which mechanism was important in creating the observed patterns, and how much of the variation in sperm motility they explained.

In summary, we found evidence of post-meiotic intra-testicular sperm senescence in *B. bufo*, and conclude that this type of sperm senescence, like other forms of sperm ageing [5], does not have a rigid, genetically fixed rate, but may also be affected by environmental conditions, such as temperature and availability of mating partners. Future studies should investigate how generally post-meiotic intra-testicular sperm senescence occurs in wild animals, what factors affect the rates of senescence and by which mechanisms they do so, and to what extent this type of sperm senescence affects reproductive behaviour, fertilization ability and offspring viability.
